# Functionalization of Electrospun Polycaprolactone Scaffolds with Matrix-Binding Osteocyte-Derived Extracellular Vesicles Promotes Osteoblastic Differentiation and Mineralization

**DOI:** 10.1007/s10439-021-02872-2

**Published:** 2021-10-18

**Authors:** Mechiel Nieuwoudt, Ian Woods, Kian F. Eichholz, Carolina Martins, Kate McSweeney, Nian Shen, David A. Hoey

**Affiliations:** 1 Department of Mechanical, Aeronautical and Biomedical Engineering, Materials and Surface Science Institute, University of Limerick, Limerick, Ireland; 2 Trinity Centre for Biomedical Engineering, Trinity Biomedical Sciences Institute, Trinity College Dublin, Dublin, Ireland; 3 Department of Mechanical, Manufacturing, and Biomedical Engineering, School of Engineering, Trinity College Dublin, Dublin, Ireland; 4 Advanced Materials and BioEngineering Research Centre, Trinity College Dublin & RCSI, Dublin, Ireland

**Keywords:** Bone, Biomaterials, Mechanobiology, Electrospinning, Polycaprolactone

## Abstract

Synthetic polymeric materials have demonstrated great promise for bone tissue engineering based on their compatibility with a wide array of scaffold-manufacturing techniques, but are limited in terms of the bioactivity when compared to naturally occurring materials. To enhance the regenerative properties of these materials, they are commonly functionalised with bioactive factors to guide growth within the developing tissue. Extracellular matrix vesicles (EVs) play an important role in facilitating endochondral ossification during long bone development and have recently emerged as important mediators of cell-cell communication coordinating bone regeneration, and thus represent an ideal target to enhance the regenerative properties of synthetic scaffolds. Therefore, in this paper we developed tools and protocols to enable the attachment of MLO-Y4 osteocyte-derived EVs onto electrospun polycaprolactone (PCL) scaffolds for bone repair. Initially, we optimize a method for the functionalization of PCL materials with collagen type-1 and fibronectin, inspired by the behaviour of matrix vesicles during endochondral ossification, and demonstrate that this is an effective method for the adhesion of EVs to the material surface. We then used this functionalization process to attach osteogenic EVs, collected from mechanically stimulated MLO-Y4 osteocytes, to collagen-coated electrospun PCL scaffolds. The EV-functionalized scaffold promoted osteogenic differentiation (measured by increased ALP activity) and mineralization of the matrix. In particular, EV-functionalised scaffolds exhibited significant increases in matrix mineralization particularly at earlier time points compared to uncoated and collagen-coated controls. This approach to matrix-based adhesion of EVs provides a mechanism for incorporating vesicle signalling into polyester scaffolds and demonstrates the potential of osteocyte derived EVs to enhance the rate of bone tissue regeneration.

**Supplementary Information:**

The online version of this article (10.1007/s10439-021-02872-2) contains supplementary material, which is available to authorized users.

## Introduction

The treatment of bone fracture in patients with compromised healing capabilities or those with severe injuries (e.g. fractures resulting in critical sized defects) represents a significant challenge to current surgical approaches and medical devices.^5^ The gold standard for bone replacement is autograft bone tissue which is limited in supply, affected by degenerative conditions, and associated with morbidity at the donor site.^56^ Tissue engineering is an approach that aims to utilise novel biomaterials to stimulate the patient’s natural healing process to promote bone regeneration and overcome the limitations of autografts. Synthetic polymeric materials have demonstrated great promise based on biocompatibility and manipulation in terms of scaffold fabrication but are limited in terms of the bioactivity when compared to naturally occurring materials. To enhance the regenerative properties of these materials, they are commonly functionalised with bioactive factors (such as BMP-2 or VEGF) to guide growth within the developing tissue.^13,31^ While these factors have demonstrated efficacy at eliciting an osteogenic and angiogenic response respectively, concerns exist regarding potential clinical side effects of these factors.^29^ An emerging approach which has the potential to deliver multiple factors simultaneously is via extracellular vesicles (EVs), where EVs are spherical membrane bound cargoes which facilitate cell–cell communication via the transfer of genetic material from one cell to another. Interestingly, EVs can bind to natural extracellular matrices (ECMs) and are known to play a role in matrix mineralisation and bone physiology,^3,18^ and thus may represent a novel approach to increase the bioactivity of synthetic materials for bone regeneration.

Microfibrous (or nano-fibrous) scaffolds are often manufactured from polyester thermopolymers such as PCL and PLA which are attractive materials due to their excellent biodegradability as well as their compatibility with a wide array of scaffold-manufacturing techniques. For example electrospinning (ES) can be used to manufacture scaffolds with well-defined mechanical properties whose structure mimics the fibrous nature of the native ECM.[^12,19,54^ Engineering the local pericellular environment in this manner provides synergistic benefits when combined with the addition of matrix proteins to the synthetic material surface or the functionalization of the scaffold with bioactive factors.^19^ Polyester materials are compatible with a wide array of chemical modifications such as adsorption, immobilization and cross-linkage.^46^ In particular, previous work by Zhu *et al*. (2002) demonstrated that both aminolysis and the 2-step crosslinkage of ECM proteins to an amine-functionalized polyester surface (via aminolysis) were effective methods for adhering matrix proteins to polyester surfaces.^57^ An interesting facet of the *in vivo* activity of bone-derived EVs is their long-observed capacity to bind to and mineralize the developing collagenous matrix during bone formation. For example, Xie *et al*. (2017) demonstrated that decalcified bone tissue could effectively bind stem cell-derived vesicles following *in vitro* soak loading and that the adhered vesicles promoted both osteogenesis and angiogenesis *in vivo* following subcutaneous implantation in a murine animal model.^55^ Therefore, functionalization of PCL surfaces with common matrix proteins (e.g. collagen type-1 or fibronectin) may provide an adhesive surface for the loading of regenerative EVs to synthetic scaffolds for bone repair.

EVs are formed from proteolipid bilayer-encapsulation of cell contents, which are then transported to, and secreted, into the extracellular space. These lipid-packaged particles carry a range of bioactive components, depending on their source and type.^48^ EVs can be broadly divided into two categories, ectosomes and exosomes. Exosomes range in size between ~ 40 and 160 nm in diameter and produced within endosomes. Ectosomes are formed via budding of the cell membrane and include microvesicles, microparticles and apoptotic bodies in the size range of ~ 50 nm up to 1 *µ*m.^30^ Vesicles play important roles throughout the body in the trafficking of important signalling molecules such as proteins, growth factors as well as mRNAs and miRNAs between cells. In bone tissue, EVs play an important role in promoting endochondral ossification via mineral crystal nucleation.^4^ As part of stimulating this process, matrix vesicles bind to the collagenous matrix within the growth plates of developing bone. Annexin-mediated Ca^2+^ channels on the vesicle surface then trigger an influx of CA ions which can lead to the nucleation of mineral crystals within the vesicle, allowing matrix mineralization in ECM regions distal to the parent cell.^21^ In addition to this important role in mineralization, recent studies have investigated mechanisms through which bone-cell derived vesicles act as important mediators of cell–cell communication within bone tissue, particularly in regulation of bone mechanoadaptation,^32,37^ a process by which bone is formed in response to mechanical loading.^7,10,44,47^ Recently Morell *et al.* (2018) demonstrated that part of osteocytes response to mechanical stimulation is the release of EVs which contain bone regulatory proteins. Moreover, Eichholz *et al.* (2020) demonstrated that that mechanically activated osteocyte derived EVs (MA-EVs) significantly enhance MSC osteogenesis.^18^ Therefore, osteocyte derived MA-EVs may represent a novel source of bioactive factors to increase the osteoinductive properties of synthetic scaffolds.

While electrospun synthetic scaffolds show promise for bone regeneration, this study aimed to enhance the regenerative properties of these materials through the surface functionalisation with ECM proteins and osteocyte derived MA-EVs, thus more closely mimicking the natural biological composition of bone. Taking into account the process through which matrix vesicles bind to developing bone matrix *in vivo*, and the *in vitro* capacity for EVs to bind a decalcified ECM, it was hypothesized that the functionalization of scaffolds with ECM proteins might provide an effective and efficient strategy for producing MA-EV functionalized scaffolds. In order to mimic the process of matrix-vesicle mineralization of collagen fibers, an electrospun mesh was used to produce a fibrous scaffold environment which, once functionalized with ECM proteins, closely resembles the topography of the collagenous native environment.^38,52^

We first optimized methods for the coating of PCL materials with collagen type-1 and fibronectin. Subsequently, we aimed to investigate the capacity for surfaces functionalized with these ECM proteins to effectively bind EVs, after which, an optimized functionalization method and adherent matrix protein were selected. Finally, an electrospun PCL scaffold was manufactured, characterized and functionalized with collagen-bound osteocyte derived MA-EVs and the capacity of this scaffold to promote osteoblast differentiation, proliferation, collagen synthesis and matrix mineralization was investigated and compared to collagen-coated and non-coated control scaffolds.

## Materials and Methods

### Polycaprolactone Membrane Preparation and Surface Modification

Sheets of polycaprolactone (PCL; Sigma Aldrich, Mn 80,000) were dissolved in chloroform at a concentration of 10 wt% and cast onto a stainless-steel plate. Following evaporation of the solvent at room temperature (RT), circular disk-membranes were punched from the sheet (8 mm diameter, 500 *µ*m thickness), and the membranes were immersed in an alcohol/water (1/1, v/v) solution for 3 h to clean the material surface, followed by a further wash with deionised water. For sodium hydroxide (NaOH) surface modification, the PCL materials were immersed in a 3 M NaOH solution for 3 h at RT and then rinsed with deionised water for 24 h. For the aminolysis surface treatment, the PCL material was immersed in a 10% (w/v) 1,6-hexamethylenediamine (HMD)/2-propanol solution for 1 h at 37 °C, rinsed with 2-propanol for 3 h and then deionised water for 24 h at RT to remove free 1,6-hexamethylenediamine.

ECM proteins, collagen or fibronectin, were attached to the PCL material (circular disks; 8 mm diameter) by (1) physical attachment or (2) immobilisation (covalent bonding) via a glutaraldehyde (GA) reaction with the aminolysed PCL surface. Following surface treatment, for the physical attachment, the PCL material was incubated in 1 mg/mL type I rat tail collagen (BD Biosciences) solution or 1 mg/mL bovine plasma fibronectin (Sigma-Aldrich) solution at 2–4 °C for 24 h. For the chemical immobilisation approach, the aminolysed PCL material were immersed in a 1 (w/v) % GA solution for 3 h at RT in order to allow NH_2_ groups to react with OHC–CHO functional groups of the GA yielding bonding via –N=CH–CHO and leaving a free aldehyde group to bond amine groups in the ECM molecules.^57^ This reaction was followed by repeated washes of deionised water for another 24 h to remove excess GA.

The material was then incubated in the 1 mg/mL collagen solution or 1 mg/mL fibronectin solution at 4 °C for 24 h. Samples were then washed twice with abundant ultrapure water, shaken for 30 min, dried at RT and stored at 4 °C.

A Pierce BCA Protein Assay Kit (Thermo Scientific) was used to quantify the protein attached to the PCL samples. Briefly, the samples were incubated in 1 mL of BCA working reagent, for 30 min at 50 °C (*n*=5). The absorbance was then measured by a spectrophotometer at 562 nm. As a reference for the collagen and fibronectin, an 8 point standard curve was calculated using stock solutions.

To measure the hydrophilicity of the samples following surface modification, water contact angles of the PCL membranes were measured at RT and 60% relative humidity (RH), using sessile drop method on a Goniometer. A distilled water drop is put on the surface of the membranes at five different sites and the average measured angle was determined (*n* = 3).

### Cell Culture

MLO-Y4 murine osteocyte-like cells were cultured on rat tail collagen (BD Biosciences, Bedford, MA) coated cell culture plastic with α-Modified Eagle's Media (α-MEM) supplemented with 5% calf serum (CS), 5% fetal bovine serum (FBS), 1% l-glutamine, and 2% penicillin–streptomycin (P/S). MC3T3-E1 (Subclone 14; ATCC, Manassas, VA) murine pre-osteoblast cells were cultured in α-MEM supplemented with 10% FBS, 1% l-glutamine and 2% P/S (all media supplements from Sigma-Aldrich unless otherwise stated). All cells were cultured under standard conditions (37 °C, 5% CO_2_).

### Isolation and Characterization of Osteocyte-Derived Mechanically Activated-Extracellular Vesicles (MA-EVs)

#### Production of Mechanically Activated Osteocyte Derived Conditioned Medium

Given the potent osteogenic properties of the mechanically stimulated osteocyte secretome,^18^ MLO-Y4 cells were seeded at a density of 80 × 10^3^ cells/well on type I collagen (0.15 mg/mL, BD) coated 6 well plates and cultured in normal growth media. 24 h following seeding, cells were washed with PBS and serum-free media was added to each well. The cells were subjected to 24 h of dynamic shear stress using an orbital shaker using the parameters set out in Salek *et al.* (2012) which produces a mean wall shear stress of approx. 0.28 Pa and a maximum wall shear stress of 1.2 Pa, a stimulation environment similar to the shear stresses used previously to produce mechanically conditioned osteocyte medium.^26,42^

#### MA-EV Isolation

EV isolation was carried out using centrifugation at 20,000 × *g* according to the protocol developed by Whitman *et al*.^51^ Briefly, the conditioned media was collected and centrifuged at 3500 × *g* for 20 min and filtered (0.45 *μ*m) to remove dead cells and cell debris. Subsequently, the supernatant was centrifuged at 20,000 × *g* for 1 h at 4 °C to pellet the MA-EVs. The supernatant was removed, and the pelleted MA-EVs were washed with PBS and pelleted again by centrifugation at 20,000 × *g* for 1 h at 4 °C. Finally, the supernatant was removed, and the pelleted MA-EVs were resuspended in PBS and used in experiments (or stored at – 80 °C for later use).

#### Transmission Electron and Confocal Imaging of MA-EVs

A single drop (~ 20 *μ*L) of the MA-EV suspension was deposited onto a carbon film grid and left to air dry for a period of 1 min. To remove excess PBS the grid was gently blotted using tissue paper through capillary action. Negative staining was achieved by placing a single drop (~ 20 *μ*L) of uranyl acetate solution onto the dry grid. Samples were imaged using a transmission electron microscope (JEOL, USA) using a voltage of 80 kV.

MA-EVs were labelled using the lipophilic dye PKH-26 Fluorescent Cell Linker Kit (Sigma-Aldrich), according to the manufacturer’s recommendations. In brief, the MA-EVs suspension was centrifuged at 20,000 × *g* for 1 h at 4 °C to pellet the MA-EVs. The pellet was incubated with 4 *μ*M PKH-26 dye solution, made up in the kit-provided diluent C for 5 min. 1 % (w/v) BSA was added to the mixture to react with the unbound dye. The suspension was centrifuged at 20,000 × *g* for 1 h at 4 °C to pellet the PKH-26 labelled MA-EVs. For the washing step the pellet was resuspended in PBS and again centrifuged at 20,000 × *g* for 1 h at 4 °C. Alternatively, for the control sample, particle-free Dulbecco's phosphate-buffered saline (DPBS; Sigma-Aldrich) was used as the input instead of the MA-EV standard. The samples were imaged with an Olympus IX83 microscope (Olympus, Hamburg, Germany) equipped with a × 100 objective (N.A. 1.40 Oil).

#### MA-EV Size Distribution Analysis

Size distribution profiles and concentration of the isolated MA-EVs were measured using the Nanosight NS300 (NanoSight Ltd.). Diluted vesicle suspensions and a PBS control were loaded into the instrument for analysis. Five 45-s videos were recorded for each sample with camera level set at 13 and detection threshold set at 5. The videos were subsequently analysed with the NTA 3.1 software which calculates the size and concentration of the particles with corresponding standard error. Auto settings were used for the analysis.

### MA-EV Attachment to Surface Functionalised PCL Membranes

The control and ECM protein immobilised PCL membranes (circular disks; 8 mm diameter) were incubated with different concentrations of PKH-26-labelled MA-EVs (low concentration = 0.3 ×10^9^ MA-EVs/mL; high concentration = 1.2 ×10^9^ MA-EVs/mL) and a dye control for 4 h at 37 °C. The samples were then washed 3 times with PBS and imaged with an Olympus IX83 fluorescent microscope. Images were analysed using ImageJ software to count positively stained vesicles.

### Electrospun Scaffold Fabrication and Functionalisation

#### Scaffold Manufacture

The electrospun scaffolds were fabricated with parameters as described in Haider *et al*.^24^. Briefly, a 12% (w/v) PCL solution in 2:1 (v/v) chloroform/methanol solution was prepared. A blunt 20-gauge stainless steel needle was used as the nozzle. A high voltage power supply was used to generate a direct current potential of 15 kV and the feed rate was controlled by syringe pump at 1.5 mL/h. The distance from the tip of the needle to the surface of the collector was a maintained at 20 cm. The collector consisted of an aluminium sheath wrapped around a rotating cylinder (ø = 10 cm) that was rotated at a speed of 300 rpm. After continuous spinning of 8 h, the thickness of the obtained PCL fibrous material was 150 *µ*m. Circular scaffolds were cut from this material using an 8 mm diameter punch. The morphology of each scaffold was investigated using a scanning electron microscope (SEM) at an accelerating voltage of 15 kV. Samples were mounted onto stubs and coated with gold using a sputter coater. The diameters of resulting nanofibers and orientation of the scaffold were analysed using ImageJ software.

#### ECM and MA-EV Functionalisation of Electrospun Scaffolds

Based on optimisation experiments on PCL membranes detailed above, collagen was identified as the optimal ECM to coat the electrospun scaffolds. Collagen was chemically immobilized to the PCL scaffold fibres as described above. The control and collagen immobilised electrospun PCL scaffolds were placed in a 48-well plate. The scaffolds were sterilised by ethylene oxide gas for 4 h at RT and then vented for 48 h to ensure that all the ethylene oxide gas was removed. To functionalize scaffolds with mechanically activated EVs, vesicles were resuspended at a concentration 1 × 10^10^ in PBS. Then sterile, collagen coated scaffolds were incubated in 1 ml of the EV-PBS solution for 4 h at 37 °C in a 48 well plate.

### *In Vitro* Biological Characterisation of Collagen and Collagen-MA-EV Functionalised Electrospun Scaffolds

#### Cell Culture and Viability Analysis

MC3T3s were seeded onto each scaffold at a density of 1 × 10^4^ cells and cultured with osteogenic medium (OM), containing EV-depleted FBS (vesicles removed via centrifugation), supplemented with 100 nM dexamethasone, 10 mM b-glycerol phosphate and 50 *µ*g/mL ascorbic acid for 21 days. The medium was changed every 3.5 days. DNA and intercellular ALP was quantified at days 1, 7, 14, and 21 (*n* = 4) and collagen and mineral production was assessed at D14 and D21 (*n* = 6).

For DNA content the samples were added to 100 *µ*L lysis buffer in 1.5 mL tubes containing 0.2% Triton X-100, 1 mM Tris pH 8, with phenyl-methylsulfonyl fluoride (PMSF) being added at a ratio of 1:200 just before use. Samples were sonicated for 60 s and subjected to three freeze–thaw cycles in liquid nitrogen before being stored on ice. DNA content was quantified using a Quant-iTTM PicoGreenTM DNA Assay Kit (Invitrogen), with excitation and emission wave-lengths of 485 nm and 528 nm respectively.

#### Intracellular ALP Analysis

Standard curves were constructed using serial dilutions of *p*-nitrophenyl phosphate (pNPP, Sigma Aldrich, N1891) with 10 *µ*L of 43 *µ*M ALP enzyme (Sigma Aldrich, P6774) in 96-well plates. Cell lysates were prepared as described above, with scaffold samples added to 100 *µ*L of a Triton–Tris lysis buffer prior to sonication and freeze–thaw cycles (× 3). 50 *µ*L of 5 mM pNPP was added to each well, with 10 *µ*L cell lysate being added followed by 70 *µ*L ddH_2_0. Samples were incubated for 1 h in the dark at RT, after which reactions were stopped using 20 *µ*L of 3 M NaOH and the plate was read at 405 nm. ALP activity was calculated as the amount of pNPP generated by samples, divided by sample volume and reaction time.

#### Collagen Production

Cell-scaffold constructs were rinsed in PBS and fixed in 10% neutral buffered formalin for 15 min before rinsing in PBS again. Scaffolds were cut in half on glass slides using 4 mm square grid paper as a guide, with the second half being used to evaluate mineral production as described later. Scaffolds were stained for 1 h while under gentle agitation (150 rpm) with 200 *µ*L of 1 mg/mL of Direct Red 80 (Sigma Aldrich, 365548) in a saturated aqueous picric acid solution, and washed twice while under gentle agitation with 0.5% acetic acid. Scaffolds were allowed to dry before imaging. They were then added to 500 *µ*L 0.5 M NaOH in 1.5 mL tubes and vortexed vigorously for 10 s to dissolve the bound stain. Tubes were centrifuged at 14,000 × *g* for 10 min to pellet the scaffold and debris. Standards were made by adding red staining solution to 8 *µ*L of collagen I (Corning, #354249), before centrifuging and re-suspending the collagen in 500 *µ*L 0.5 M NaOH. Samples and standards were added to a 96-well plate and the absorbance read at 520 nm. Quantification of calcium production was calculated as increased calcium over the baseline detected in cell-free PCL or PCL-collagen controls.”

#### Mineral Production

Alizarin red solution was made at a concentration of 10 mg/mL of alizarin red S (Sigma Aldrich, A5533) in distilled water, with the pH being adjusted to between 4.1 and 4.3. 200 *µ*L of alizarin red solution was added to each sample and shaken for 20 min. Samples were then washed 5 times for 3 min with shaking in distilled water and allowed to dry before imaging. Wells containing the stained scaffolds were then filled to 400 *µ*L with 55% acetic acid and the solution transferred into 1.5 mL tubes and incubated for 18 h with 150 rpm shaking at RT. Tubes were vortexed vigorously for 30 s, heated to 85 °C for 1 h, placed on ice for 5 min and then centrifuged at 20,000 × *g* for 15 min to pellet the scaffold and debris. 300 *µ*L of supernatant was transferred to new tubes and 120 *µ*L of ammonium hydroxide added. Standards were made with known dilutions of alizarin red solution in water with the pH for each standard adjusted to between 4.1 and 4.3. Samples and standards were added to a 96-well plate and the absorbance read at 405 nm. Quantification of calcium production was calculated as increased calcium over the baseline detected in cell-free PCL or PCL-collagen controls.

### Statistical Analysis

All data is presented in terms of average and standard deviation. Statistical analysis was performed using One-way and Two-way ANOVA as appropriate with a Tukey’s post-test to compare means across treatment groups. Statistics were preformed using GraphPad Prism v8 software package. Significance was accepted at a level of *p* < 0.05.

## Results

### Surface Modification and Biocompatibility of PCL Membranes

**Figure 1:**
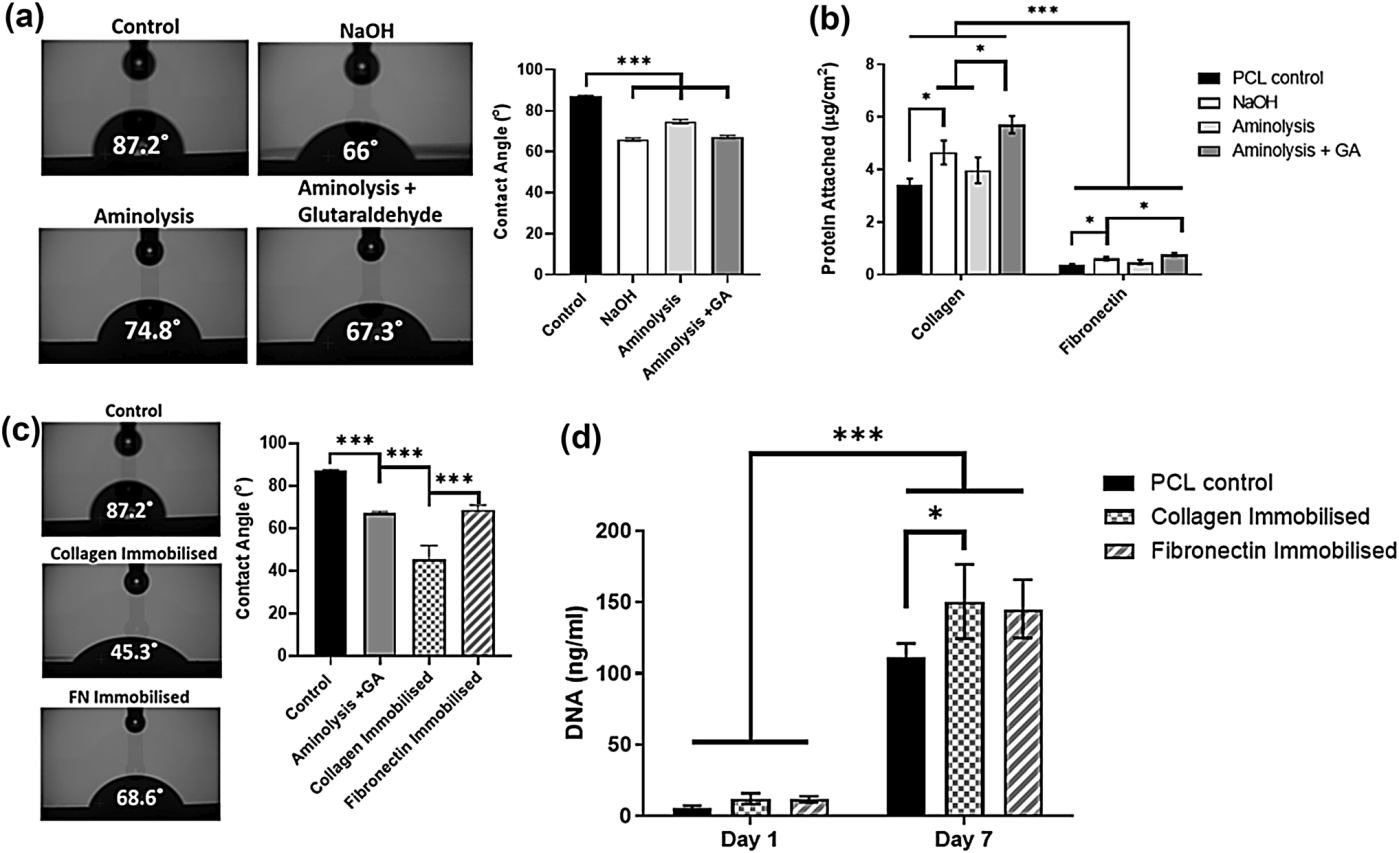
Comparison of surface modification on ECM attachment, hydrophilicity, cell adhesion, and proliferation. **(a)** Hydrophilicity analysis of modified PCL membranes and quantification of water contact angle. **(b)** BCA analysis of the quantity of ECM protein attachment to the PCL membrane surface. **(c)** Water contact angle analysis and quantification of the effect of protein immobilisation on PCL membrane hydrophilicity. **(d)** Cell number quantification 24 h after seeding, as an indicator of initial cell adhesion, and after 7 days and an indicator of cell proliferation. All data *n* = 3–6. Statistical analysis using one‐way or two-way analysis of variance (ANOVA) and Tukey's multiple comparison post‐test as appropriate (*p < 0.05, ***p < 0.001).

PCL membrane surfaces was successfully modified using NaOH treatment, aminolysis and immobilization (aminolysis following by GA) treatment (Fig. 1) resulting in a significant decrease in membrane hydrophilicity (*p* < 0.001) measured using water contact angle analysis (Fig. 1a). Following incubation of the treated surfaces with collagen or fibronectin, BCA analysis (Fig. 1b) indicated that NaOH treatment and immobilization resulted in a significant (*p* < 0.05) increase in collagen and fibronectin attachment to the modified surface compared to PCL controls although no significant increase in protein attachment was observed in the aminolysis group. The collagen immobilised group exhibited the best collagen attachment (5.71 ± 0.17 *μ*g/cm^2^), 69% better than the control (3.4 ± 0.26 *μ*g/cm^2^; *p* < 0.001) and it was also significantly better than the other surface modified groups. Similarly, the fibronectin immobilised group gave 106% better attachment (0.76 ± 0.03 *μ*g/cm^2^; *p* < 0.001) than the control. The collagen immobilised group had 7.5-fold (*p* < 0.001) more protein attached than the fibronectin immobilised group and exhibited significantly increased (*p* < 0.001) hydrophilicity (Fig. 1c) compared to non-collagen treated surfaces. Analysis of cell attachment at 1 day post seeding and changes in cell number over a 7-day culture period (Fig. 1d) indicated that cells readily attached to and proliferated on the modified substrates and the modified groups trended towards increased attachment and total cell number. A significant increase (*p* < 0.05) in total cell number was observed on the collagen immobilised group when compared to PCL controls after 7 days.

### MA-EV Characterization and PCL Surface Adhesion

**Figure 2:**
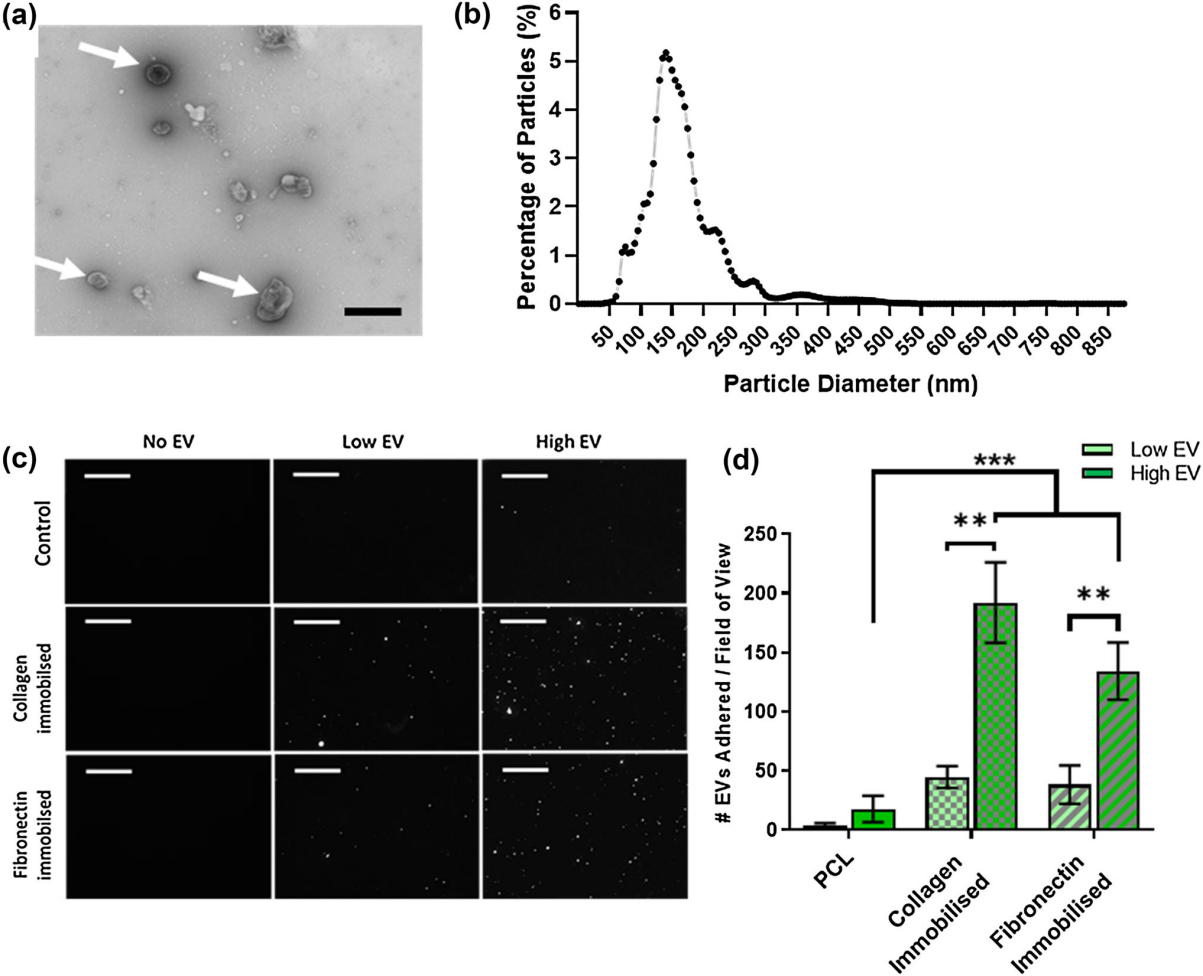
Characterization of MA-EVs and functionalisation of PCL membranes. **(a)** TEM analysis of MA-EV morphology, showing presence of collapsed spheres indicative of EV presence (arrows) within isolates (scale bar = 500 nm). **(b)** Nanoparticle size analysis of MA-EVs measuring a majority of particles within the range of 60–300 nm, indicative of a mixed population of exosomes and microvesicles. **(c** and **d)** Confocal immunofluorescent images and quantification of PKH-26 labelled EVs attached to functionalised PCL membranes (scale bar = 10 *µ*m). All data *n* = 3. Statistical analysis using two-way analysis of variance (ANOVA) and Tukey's multiple comparison post‐test (**p < 0.01, ***p < 0.001).

TEM analysis of MA-EVs isolated from MLO-Y4 conditioned medium (Fig. 2a) showed the characteristic double lipid membrane “collapsed-bowl” structure indicating that vesicles were present within the conditioned medium (arrows). This is consistent with previous work, which utilised ultracentrifugation to isolate EVs from MLO-Y4 conditioned medium.^18^ Nanoparticle size analysis (Fig. 2b) indicated that the majority of particles within the conditioned medium were within the range of 60–300 nm, indicating a mixed population of microvesicles and exosomes within the EV isolate.

Analysis of the adherence of PKH-26 labelled EVs to functionalized PCL membranes Figs. 2d and 2e indicated an EV-concentration dependent effect of the quantity of labelled EVs adherent on the membranes. Membranes incubated with a higher concentration of EVs (1.2 × 10^9^ MA-EVs/mL) exhibited a significant 5-fold and 4-fold increase (*p* < 0.01) in EV attachment on collagen or fibronectin functionalized membranes respectively, over surfaces incubated with a “low” concentration (0.3 ×10^9^ MA-EVs/mL). Furthermore, both protein functionalized surfaces exhibited a significant increase in EV attachment (*p* < 0.001) indicating that ECM-protein functionalization is an effective way of adhering EVs to a PCL surface. No significant difference in EV attachment was observed between either protein type, despite significantly less fibronectin being functionalized to the surface. As such, lower concentrations of fibronectin may be effective at binding vesicles at an increased rate compared to similar concentrations of collagen type-I.

### Electrospun Scaffold Characterization and MA-EV Functionalization

**Figure 3:**
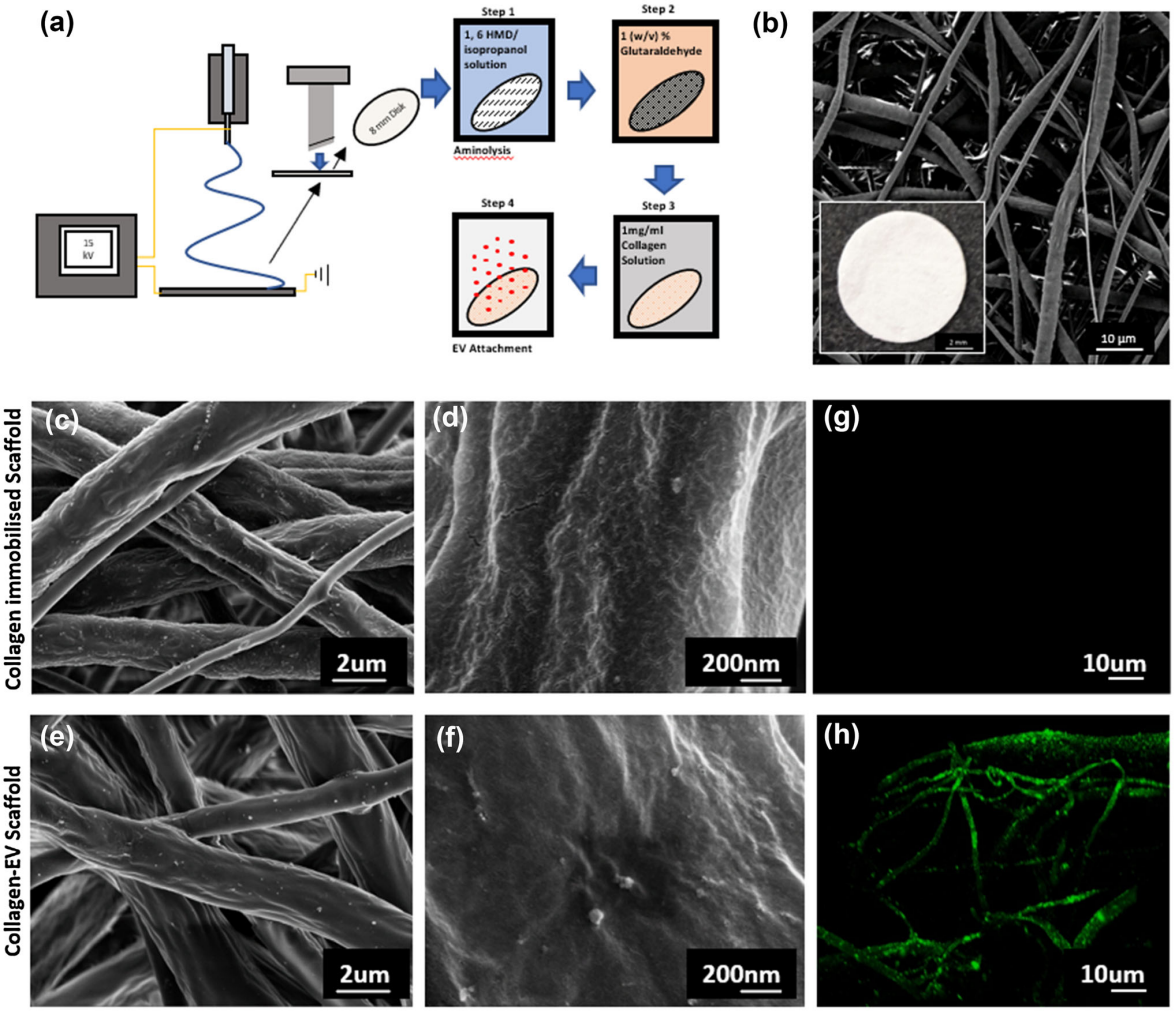
Electrospun scaffold characterization, functionalization and EV attachment. **(a)** Schematic diagram of electrospinning and functionalization process. **(b)** SEM image of 8 mm electrospun PCL scaffold (inset: macro image of scaffold). **(c–f)** SEM imaging of scaffold fibre morphology of collagen functionalised and collagen-EV functionalised scaffolds respectively. **(g** and **h)** Confocal imaging of collagen-functionalized scaffold after incubation with a PKH-26 dye control or PKH-26 labelled EVs respectively.

Electrospun scaffolds were fabricated and functionalised with collagen and MA-EVs as detailed in Fig. 3a. The macro-appearance of the 8 mm PCL scaffolds used throughout the study is illustrated in Fig. 3b. SEM analysis of the fibrous microstructure indicated a microfiber morphology with a smooth PCL fibre surface with an average fibre diameter of 1.47 ± 0.6 *µ*m. Following collagen-immobilization, fibres exhibit a slightly rougher, scaled appearance (Figs. 3c and 3d). Following further incubation with MA-EVs, numerous spherical features are visible across the PCL fibre (Figs. 3e and 3f). It is unclear if these are EVs as similar features were found in the collagen alone samples, although at a much lower frequency. To further verify the functionalisation of EVs onto electrospun PCL fibres, confocal imaging of the binding of PKH-26 labelled EVs on the scaffold surface was completed. As shown in Fig. 3h, the electrospun fibres are decorated with the PKH-26 indicating successful attachment of MA-EVs when compared to scaffolds incubated with a dye control (Fig. 3g).

### MA-EV Functionalisation Promotes Osteoblast Differentiation and Increases the Rate of Matrix Mineralization

**Figure 4:**
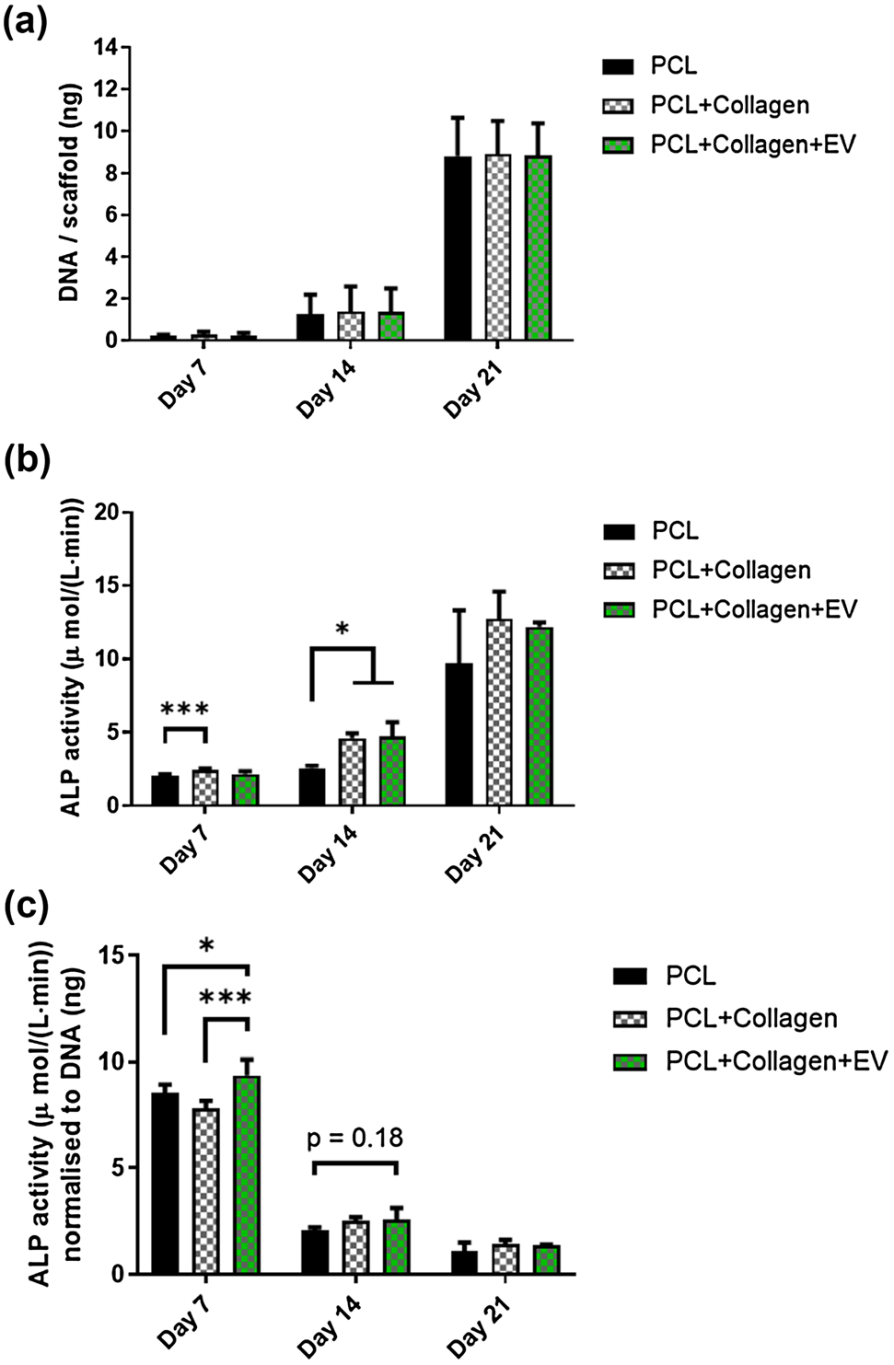
Cellular proliferation and ALP activity of MC3T3 cells cultured on PCL, PCL + Collagen, and PCL + Collagen + EV scaffolds. **(a)** Quantification of DNA content of scaffolds at 7, 14, and 21 days post seeding. **(b)** Quantification of total ALP activity at 7, 14, and 21 days and **(c)** ALP activity normalised to DNA content per scaffold. All data *n* = 4. Statistical analysis using two-way analysis of variance (ANOVA) and Tukey's multiple comparison post‐test (**p < 0.01, ***p < 0.001). *ALP* alkaline phosphatase, *EVs* extracellular vesicles.

Collagen coating and further EV functionalisation of PCL electrospun scaffolds does not influence osteoblast proliferation but enhances early osteogenesis. Analysis of the DNA content of the scaffolds at 7, 14, and 21 days demonstrated a significant increase in osteoblast proliferation over the 3 weeks although no significant differences in cellular attachment or proliferation were found between the PCL, PCL + Collagen or PCL + Collagen + EV scaffolds (Fig. 4a). Significant changes in total ALP activity were observed in PCL + Collagen samples after 7 days (compared to PCL control). After 14 days both the PCL + Collagen and PCL + Collagen + EV scaffolds exhibited a significant increase in ALP activity compared to PCL alone. This difference is lost at 21 days (Fig. 4b). Similar trends are evident when ALP activity was examined on a per-DNA basis, although the significance is lost at 14 days (Fig. 4c). Taken together this data indicates that collagen coating and further functionalisation with EVs has the potential to enhance early osteogenesis.

**Figure 5:**
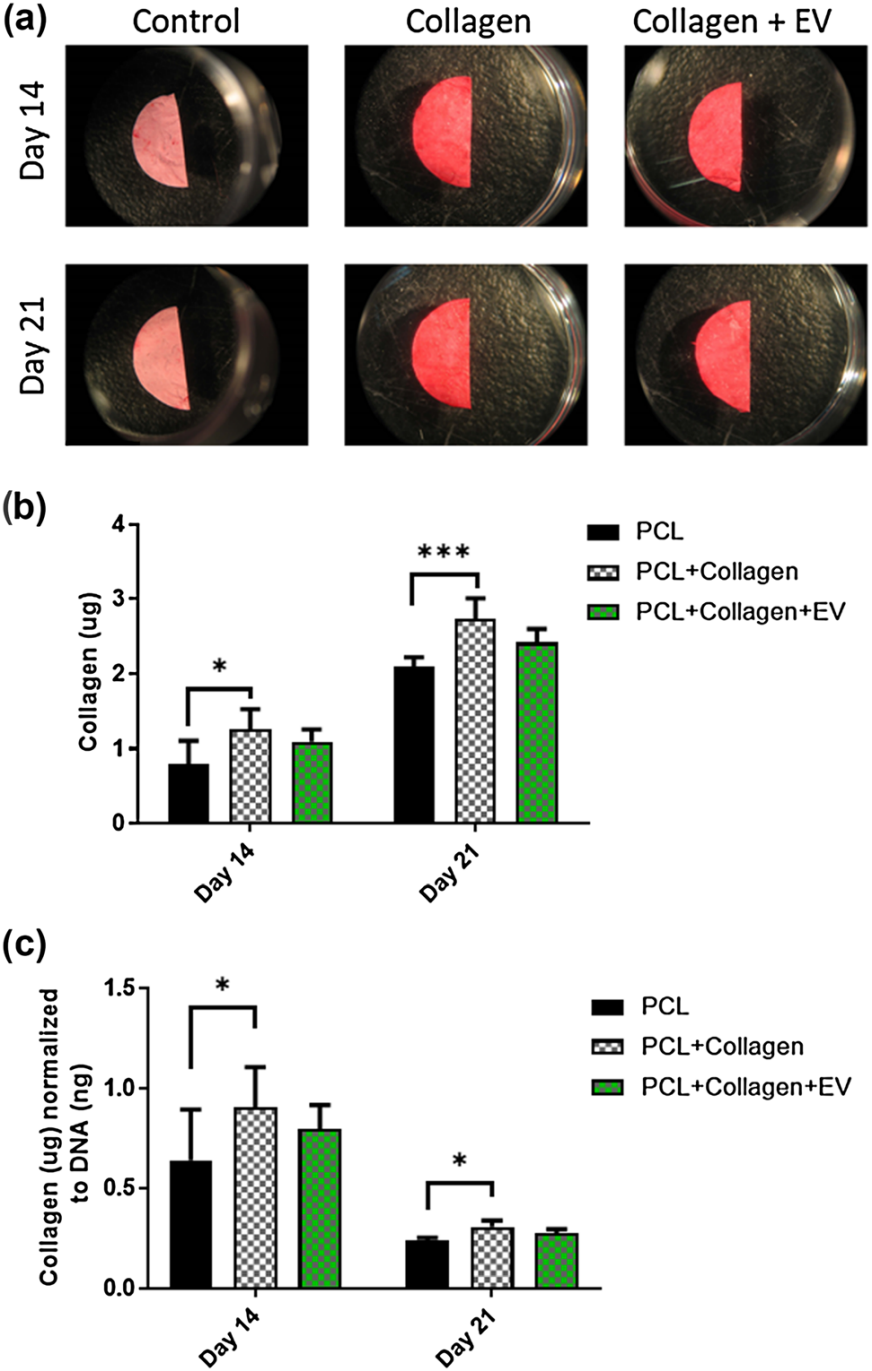
The effects of PCL scaffold surface modifications on collagen extracellular matrix deposition. **(a)** Picosirius red staining of PCL, PCL + Collagen, and PCL + Collagen + EV scaffolds after 14- and 21 days culture. **(b)** Quantification of collagen matrix after 14 and 21 days. **(c)** Quantification of collagen synthesis normalised to DNA content after 14 and 21 days. All data *n* = 6. Statistical analysis using two-way analysis of variance (ANOVA) and Tukey's multiple comparison post‐test (*p < 0.05, ***p < 0.001). *EVs* extracellular vesicles.

**Figure 6:**
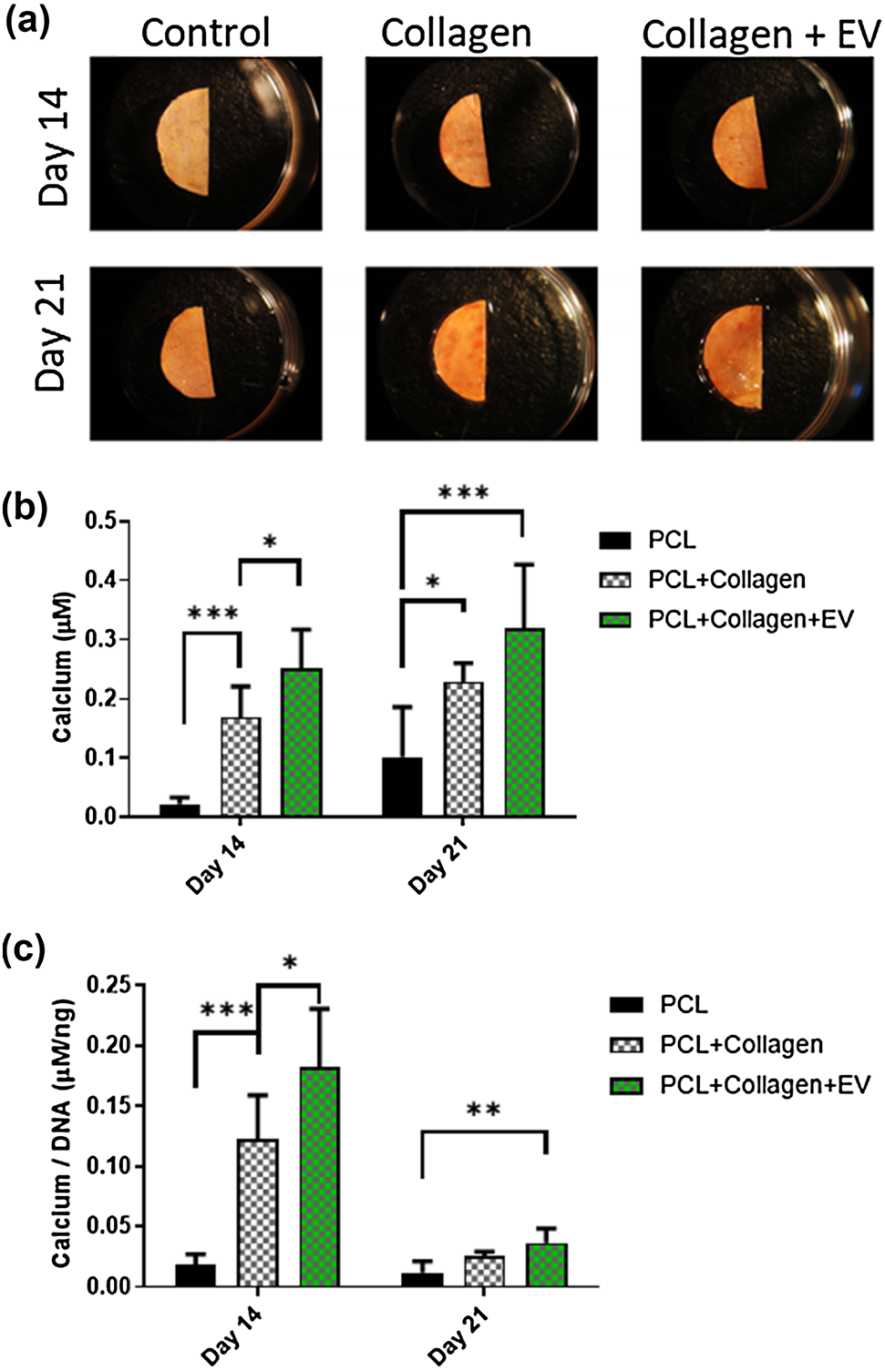
The effects of PCL scaffold surface modifications on mineral deposition. **(a)** Alizarin red staining of PCL, PCL + Collagen, and PCL + Collagen + EV scaffolds after 14- and 21 days culture. **(b)** Quantification of calcium after 14 and 21 days. **(c)** Quantification of calcium synthesis normalised to DNA content after 14 and 21 days. All data *n* = 6. Statistical analysis using two-way analysis of variance (ANOVA) and Tukey's multiple comparison post‐test (*p < 0.05, **p < 0.01, ***p < 0.001). *EVs* extracellular vesicles.

To determine if this early increase in ALP activity correlated to enhanced osteogenic matrix deposition, we next examined collagen and calcium deposition after both 14 and 21 days. Both the PCL + Collagen and PCL-Collagen-EV scaffolds stain more vividly with picrosirius red indicating an enhanced presence of collagen (Fig. 5a). While this is not unexpected given the collagen coating, quantification of this labelling demonstrated that the total amount of collagen increased over time indicating a cell mediated deposition of the ECM protein. Interestingly only the PCL-Collagen scaffold outperformed the PCL control, indicating the further EV functionalisation may hinder collagen production (Fig. 5b). Similar to Collagen deposition, increased alizarin red staining was visible on PCL-Collagen and PCL-Collagen-EV scaffolds after 21 days (Fig. 6a). Quantification of total mineral content demonstrated a significant increase in calcium deposition following collagen coating of the scaffolds (7.7-fold, 14 days, *p* < 0.001; 2.1-fold, 21 days, *p* < 0.05), which was further significantly enhanced following EV functionalisation of the PCL fibres after 14 days of culture, and this trend continued after 21 days, although the difference was not significant (2.1-fold, 14 days, *p* < 0.05; 1.4-fold, 21 days) (Fig. 6b). A similar trend in calcium deposition is present when normalised to DNA content indicating that EV functionalization of the PCL surface promotes earlier mineralization of the matrix by seeded MC3T3 cells leading to an overall improvement of mineralized matrix deposition (Fig. 6c)

## Discussion

Harnessing the growing understanding of vesicle-based signalling for use in bone tissue applications requires tools and protocols that enable their incorporation into innovative scaffold designs. In this paper, we optimize a method for the functionalization of PCL materials with collagen type-1 and fibronectin, inspired by the behaviour of matrix vesicles during endochondral ossification, and demonstrate that this is an effective method for the adhesion of EVs to the material surface. We then used this functionalization process to attach osteogenic osteocyte derived MA-EVs to collagen-coated electrospun PCL scaffolds. The MA-EV-functionalized scaffold promoted osteogenic differentiation (measured by increased ALP activity) and mineralization of the matrix. In particular, MA-EV-functionalised scaffolds exhibited significant increases in matrix mineralization on a per-cell basis at earlier time points compared to collagen-coated controls while also exhibiting a trend towards higher overall mineral content. This approach to matrix-based adhesion of EVs provides a mechanism for incorporating vesicle signalling into polyester scaffolds and indicates that osteocyte derived MA-EVs can provide effective osteogenic signalling which stimulates the *in vitro* mineralization of cell seeded scaffolds.

In the first part of the study, the effect of hydrolysis, aminolysis and immobilization on PCL surface wettability and protein attachment were shown to all significantly improve the hydrophilicity of the PCL membrane, indicating an increase in the surface energy of the material (and significant changes to the material surface), as has been observed in previous studies.^11,17,45,57^ Furthermore, the levels of matrix protein attachment compared favourably to other work—for example Zhu *et al*. (2009) demonstrated similar levels of collagen-IV attachment to aminolysis-modified PCL surfaces.^58^ The hydrolysed surfaces, with increased surface charge, exhibited a significant increase in both collagen and fibronectin attachment compared to the aminolysed surface, an expected result as the binding of proteins to both of these surfaces occurs mainly via electrostatic and Van Der Waals forces. However, the use of GA to covalently couple proteins to the surface resulted in the highest increase in protein attachment of all groups, likely due to the change adhesion mechanism to a covalent bond instead of electrostatic interactions. Protein adsorption to surfaces is a commonly occurring but difficult to study phenomena and changes in the conformation and denaturation of proteins on material surfaces during adsorption can result in strong and effective attachment of proteins to biomaterial surfaces.^39^ Covalent conjugation, in contrast, allows adhesion to occur more rapidly, without necessitating denaturation and the unfolded proteins can be more densely and efficiently adhered to the material surface. The process is mediated through the addition of a free aldehyde group to the amine-treated surface and allows the binding of matrix proteins following extensive washing, diminishing the risk of cytotoxic GA residues^22^ or necessity of post treatment with a GA-quenching glycine treatment while also avoiding direct contact of matrix proteins with the crosslinking solution which helps to retain their bioactivity, which chemical crosslinking can inhibit.^23^ The excellent biocompatibility of the membranes and the electrospun scaffolds serve to reinforce this point, with protein-immobilized surfaces enhancing cellular attachment and proliferation. The immobilization process resulted in significantly more collagen than fibronectin attachment to the membranes. While collagen surfaces exhibited the best capacity to bind EVs, the collagen-functionalized PCL only exhibited a non-statistically significant 30% increase in vesicle attachment despite a 7.5-fold higher mass of collagen being adhered to the membrane surface. This difference in efficiency indicated that fibronectin may be a more generally adhesive substrate for vesicle adhesion but due to low affinity to the PCL substrate, proved less effective overall as an adherent coating for vesicles. This may be due to differing levels of affinity and mechanisms of binding between vesicles and different matrix proteins.^2,8,9^ For example, tissue non-specific alkaline phosphatase (TNAP), several members of the calcium binding Annexin membrane protein family (Annexins A2, A5 and A6) as well as several matrix-protein binding integrins are known the be expressed on the surface of bone cell derived vesicles.^6,9,53^ Interestingly, Annexin-A5, the expression of which is upregulated in mineralizing vesicles, has been shown to be essential for the binding of proteoliposomes to collagen fibrils.^15,33,49^ Furthermore, collagen and fibronectin binding integrin *α*_v_*β*_3_ is also expressed on the surface of bone cell-derived EVs and recent work by, Altei *et al*. (2020) indicates that its presence may be essential for binding of breast cancer cell-derived vesicles to fibronectin.^1^ Further evidence of the essential role of integrins in binding vesicles to matrix proteins has recently been published by Hao *et al*. (2020) who demonstrated that functionalization of a material surface with a LLP2A, an integrin *α*_4_*β*_1_ ligand to which placental MSCs exhibited high affinity, could be used to effectively bind MSC-derived EVs and promote angiogenesis.^25^ In summary this data demonstrates that harnessing the matrix binding properties of EVs is an effective method to functionalise synthetic polymeric materials and that collagen type-1 immobilized to the PCL surface was an effective surface treatment for binding osteogenic vesicles.

Following the successful adherence of EVs to collagen coated PCL membranes, the same immobilization process was applied to electrospun microfiber PCL scaffolds. Osteocyte derived MA-EVs were produced using an orbital shaker to apply oscillating shear flow to the cultured osteocytes, an approach that has been shown to deliver forces within a range that stimulates osteocyte and epithelial cell mechanosignalling.^14,26,27^ Fluorescently labelled MA-EVs were found to decorate the surface of fibres demonstrating that the collagen-coated PCL microfibers acted as an adherent surface for EVs. As expected, the addition of the collagen coating to the PCL fibers resulted in a trend towards increased cellular proliferation in seeded MC3T3-E1 cells, as previously observed in collagen coated membranes, and significantly increased ALP expression, collagen synthesis and matrix mineralization relative to the non-coated controls. The pro-osteogenic effects of collagen type-1 on osteoblast cells in general, and MC3T3 cells in particular are well known. For example, Lynch *et al*. (1995) cultured rat calvarial osteoblasts on collagen-I films and found that osteoblasts exhibited increased expression of ALP following 7 days of culture, as well as significantly enhanced proliferation and cell-normalized calcium content following 14 days of culture and beyond—trends similar to those observed in this study. Similarly, Liu *et al*. (2014) demonstrated that the treatment of MC3T3-E1 cells with bovine collagen peptides promoted increased ALP gene expression following 7 days of culture and RUNX-2 gene expression following 14 days of culture as well as significantly enhanced matrix mineralization following 14 days of culture.^35,36,50^ The addition of MA-EVs to the scaffold surface resulted in a significant increase in ALP activity on a per-cell basis which matches observations we have previously published on the effect of MA-EVs in promoting osteogenic differentiation in human bone marrow stem/stromal cells in 2D.^18^. Furthermore, matrix mineralization was significantly increased on a per-cell basis at later time points and mineralization trended higher in MA-EV functionalised scaffolds throughout the study, providing evidence that MA-EVs promote osteoblastic stem cell differentiation and matrix mineralization, and thus represent an alternative bioactive factor to functionalise scaffolds for bone repair.

Osteocyte (and MLO-Y4)-derived EVs have been shown to contain a variety of mRNAs and miRNAs implicated in regulating osteogenic differentiation. For example, Qin *et al*. (2017) recently demonstrated that mineralization promoting skeletal stem cell-derived EVs were highly enriched with miR-16a, miR-27a and miR-206, each of which are critical for osteogenesis.^41,43^ At the same time, the role that vesicles play in mineral crystal nucleation is also likely to promote matrix mineralization. Recent studies have begun to examine the role of vesicles in acellular matrix mineralization for tissue engineering applications.^16,41,55^ Davies *et al.* (2017) has shown that Annexin-enriched vesicles extracted from osteoblast cultures significantly enhanced *in vitro* mineralization while Furuta *et al*. (2016) have demonstrated that intravenous injection of skeletal stem cell derived vesicles promote *in vivo* regeneration in a CD9^−/−^ murine model of fracture repair possessing diminished vesicle synthesis.^15,20^ Given that our previous work has indicated that the osteocyte-conditioned medium, from which the MA-EVs are derived, exhibits Annexin-enrichment, it therefore seems likely that the observed increase in matrix mineralization is a combination of both cellular uptake and mineral crystal nucleation.^18^

While the study successfully demonstrates that collagen type-1 and fibronectin coating of a PCL microfiber scaffold enables the attachment of osteogenic MA-EVs, the study possesses several limitations. First, while we have previously characterized osteocyte-derived MA-EVs in more detail, the change in stimulatory regime from parallel flow plate to orbital shaker may have changed the nature of the mechanosigalling conveyed by the EVs, as several studies have demonstrated that the osteocyte mechanosignalling is dependent on the size and nature of the applied mechanical stimulus.^34,40^. Second, as observed in the results and highlighted earlier, vesicles exhibit protein specific binding affinities, and while collagen type-I was chosen due to its ubiquity within bone tissue and its known compatibility with osteoblast culture, future work which examines functionalization of scaffolds with alternative types of collagen fibril may increase capacity of the functionalized surface to bind EVs or potentially select for specific EV populations which preferentially bind to collagen. Furthermore, MC3T3-E1 cells possess limitations in their capacity to accurately model matrix mineralization or bone formation with significant differences in gene expression observed between these osteoblast-like cells and primary osteoblasts.^28^. Lastly, while we have demonstrated enhanced matrix mineralization with MA-EV functionalisation, we can at this time only speculate as to the mechanisms at play here. Further work exploring the contents of these EVs may reveal more specific targets to enhance bone regeneration.

In conclusion, ECM protein attachment was demonstrated to be an effective means of functionalizing PCL surfaces with bioactive EVs. Immobilization was shown to be more effective than either hydrolysis or aminolysis for coating the PCL surface with matrix proteins and the collagen surface was shown to be the most effective at binding EVs, although comparatively, fibronectin was more effective on a per gram basis of protein attached. The same approach was shown to successfully coat an electrospun PCL-collagen scaffold with osteocyte derived MA-EVs and the presence of these MA-EVs significantly enhanced osteogenesis within the scaffold. The technique we present is applicable not only to electrospun scaffolds, but also to a wide range of scaffold manufacturing techniques that use PCL or other polyesters, such as melt electro-writing or 3D printing and offers guidance for future work seeking to equip synthetic polymer tissue engineering scaffolds with EVs.

## Supplementary Information

Below is the link to the electronic supplementary material.

(MP4 3912 kb)
